# A Patient-Screening Tool for Clinical Research Based on Electronic Health Records Using OpenEHR: Development Study

**DOI:** 10.2196/33192

**Published:** 2021-10-21

**Authors:** Mengyang Li, Hailing Cai, Shan Nan, Jialin Li, Xudong Lu, Huilong Duan

**Affiliations:** 1 College of Biomedical Engineering and Instrument Science Zhejiang University Hangzhou China; 2 Key Laboratory for Biomedical Engineering Ministry of Education Hangzhou Zhejiang China; 3 Hainan University School of Biomedical Engineering Haikou City China

**Keywords:** openEHR, patient screening, electronic health record, clinical research

## Abstract

**Background:**

The widespread adoption of electronic health records (EHRs) has facilitated the secondary use of EHR data for clinical research. However, screening eligible patients from EHRs is a challenging task. The concepts in eligibility criteria are not completely matched with EHRs, especially derived concepts. The lack of high-level expression of Structured Query Language (SQL) makes it difficult and time consuming to express them. The openEHR Expression Language (EL) as a domain-specific language based on clinical information models shows promise to represent complex eligibility criteria.

**Objective:**

The study aims to develop a patient-screening tool based on EHRs for clinical research using openEHR to solve concept mismatch and improve query performance.

**Methods:**

A patient-screening tool based on EHRs using openEHR was proposed. It uses the advantages of information models and EL in openEHR to provide high-level expressions and improve query performance. First, openEHR archetypes and templates were chosen to define concepts called simple concepts directly from EHRs. Second, openEHR EL was used to generate derived concepts by combining simple concepts and constraints. Third, a hierarchical index corresponding to archetypes in Elasticsearch (ES) was generated to improve query performance for subqueries and join queries related to the derived concepts. Finally, we realized a patient-screening tool for clinical research.

**Results:**

In total, 500 sentences randomly selected from 4691 eligibility criteria in 389 clinical trials on stroke from the Chinese Clinical Trial Registry (ChiCTR) were evaluated. An openEHR-based clinical data repository (CDR) in a grade A tertiary hospital in China was considered as an experimental environment. Based on these, 589 medical concepts were found in the 500 sentences. Of them, 513 (87.1%) concepts could be represented, while the others could not be, because of a lack of information models and coarse-grained requirements. In addition, our case study on 6 queries demonstrated that our tool shows better query performance among 4 cases (66.67%).

**Conclusions:**

We developed a patient-screening tool using openEHR. It not only helps solve concept mismatch but also improves query performance to reduce the burden on researchers. In addition, we demonstrated a promising solution for secondary use of EHR data using openEHR, which can be referenced by other researchers.

## Introduction

Clinical research is a scientific research activity that considers patients as the main research object and focuses on the diagnosis, treatment, and prognosis of diseases. The identification of research subjects during clinical research is one of the major challenges. A study [1] in Britain showed that of the 114 surveyed clinical studies, only 35 (31%) could complete patient screening as planned. During the design of the research protocol, researchers develop detailed conditions for eligible patients. In the past, researchers collected eligible patients by asking clinicians or manually issuing recruitment ads. However, this is a labor-intensive and time-consuming task and can be helpful in small clinical research. The widespread adoption of electronic health records (EHRs) has enabled the secondary use of EHR data for clinical research.

However, there exist many obstacles to be overcome in using EHR data for clinical research. Fragmentation of clinical data and proprietary health information systems make it a challenge to adopt some specific screening methods [2-6]. These methods require detailed communication among researchers, clinicians, and information technology personnel each time. So, it is a time-consuming and error-prone process due to communication errors [7]. Only a few EHR vendors adopt health information standards and accommodate controlled terminologies [8]. Researchers have to express their query requirements into keywords to select patients from EHRs [9-12]. Due to these conditions, query tools based on EHRs are required for clinical research. Form-based query interfaces, such as Informatics for Integrating Biology & the Bedside (i2b2) [13], provide a promising direction for queries on EHRs. These interfaces partially meet query requirements by providing controlled query inputs for built-in coded concepts. However, complex screening conditions cannot be effectively and accurately expressed this way, especially for derived concepts. Wagholikar et al [14] proposed that derived concepts can only be expressed by Structured Query Language (SQL), which is a challenging task. The lack of domain-specific high-level expression of SQL makes it difficult for researchers to express these derived concepts. In addition, these query interfaces, such as i2b2, are mostly treated as clinical data warehousing and store EHR data through the star model. When faced with subqueries and join queries, query tools based on relational databases are inefficient [15].

Accordingly, a user-centered patient-screening tool with high-level expressions based on a standardized and scalable clinical data repository (CDR) can facilitate the use of EHR data in clinical research. OpenEHR is regarded as a promising tool to help build a CDR and support the expression of complex screening conditions. It provides a new formal modeling paradigm from clinical contents [16]. Its several features make it attractive in helping build patient-screening tools for clinical research. First, it provides open, semantically enabled, standard-based, vendor-independent, and use-case agnostic information models to represent clinical concepts [17]. It reuses existing archetypes in many particular clinical use cases across templates to reduce time and effort to enable semantic interoperability among different systems. This feature lays a solid foundation for the development of a CDR. Some studies on openEHR-based CDRs have been proposed [18-20]. Second, openEHR divides models into the archetype model (AM) and the reference model (RM). The AM can be used to represent domain knowledge. Within the AM, many coded values set or coding vocabularies can be drawn from controlled terminology resources [21-25]. Engineers only need to focus on developing software based on the RM, which facilitates maintainability [26]. This way, developers can provide different implementations for specific requirements. In addition, it provides openEHR openEHR Expression Language (EL) [27] to specify archetype rules and decision expressions. OpenEHR EL can be used to represent high-level query expressions combined with coding concepts. Domain-specific languages have shown promise in many use cases. So, openEHR EL makes it possible for clinical researchers to query on EHRs compared to SQL.

However, it is still an open question how openEHR can be applied in patient screening for clinical research. Specifically, two questions need to be tackled, the lack of high-level expressions and inefficient queries. Accordingly, in this study, openEHR EL is used to provide high-level expressions for queries, especially for derived concepts. Meanwhile, inefficient queries are generated for these derived concepts because they consist of simple concepts and complex constraints. Therefore, Elasticsearch (ES) [28] is introduced to build the underlying CDR for patient screening. By generating hierarchical indexes for corresponding archetypes and templates, our method avoids executing join queries and subqueries. To the best of our knowledge, there are almost no such query tools aimed at complex medical concepts in openEHR-based CDRs using ES.

The structure of the rest of this paper is as follows. Our method is proposed in the Materials and Methods section. After query requirements are collected, a representation method is proposed for eligible criteria based on archetypes and openEHR EL. Afterward, ES is used to generate hierarchical indexes based on archetypes. Finally, a screening tool is developed to support patient-screening tasks. The Results section gives the screening condition representation and execution performance evaluation. The Discussion section describes the contributions of our method and some relevant issues and future directions. Finally, conclusions are summarized.

## Methods

### Requirement Collection

Since it is difficult to collect actual query requirements in clinical research due to fragmented requests, conflicts of interest, security, etc, we considered clinical trials as representative examples of clinical research. A clinical trial is an experiment designed to answer specific questions about possible new treatments or new ways of using existing (known) treatments. To analyze the requirements of screening in clinical research, 389 stroke-related clinical trials were collected up to January 1, 2020, from the Chinese Clinical Trial Registry (ChiCTR) [29], including 2178 inclusion criteria and 2513 exclusion criteria, with a total of 4691 screening criteria. All these criteria were considered as the query requirements in this paper.

### Representation of Screening Conditions

One of the major functions of patient-screening tools is to transform screening conditions in free text into computer-readable expressions. Ross et al [30] analyzed the composition and structure of screening conditions. Weng et al [31] surveyed the formal representation of eligibility criteria in clinical trials. Many representation methods in these studies can be used. One of the main considerations in the development of patient screening is to make it compatible between the representation of screening conditions and data representation in EHRs.

OpenEHR is proposed to represent the data structure in EHRs. EL is part of the openEHR specification for specifying archetype rules and decision language expressions. OpenEHR EL is based on the openEHR information model and is consistent with the structure in EHRs. Therefore, our study uses openEHR EL to represent screening conditions. OpenEHR EL provides complete arithmetic operators, relational operators, logical operators for different kinds of operations, and limited operations about time and collections. These operators do not meet the requirements of complex screening conditions in some cases (eg, for patients who meet the requirements of “white blood cell count continues to decrease in a specific duration after chemotherapy and radiation”).

Consequently, for representing screening conditions, our method can be divided into two parts:

1. Defining concepts from openEHR archetypes and templates directly
2. Generating concepts by openEHR EL according to clinical requirements

**Figure 1:**
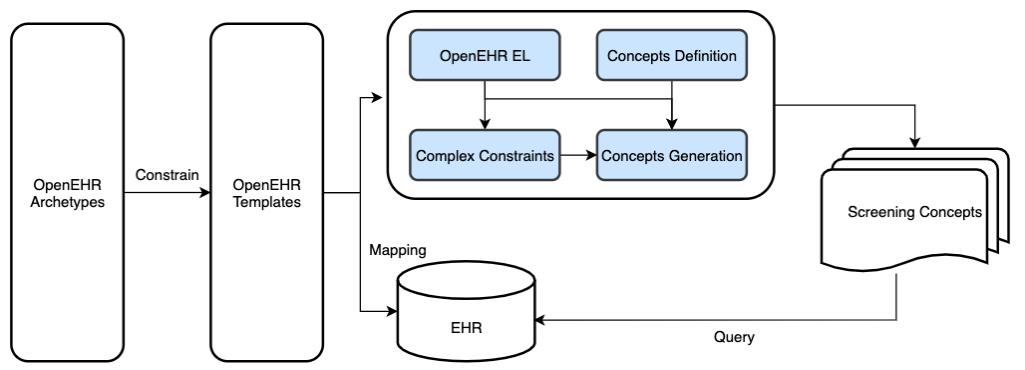
A process of representation for screening conditions. EHR: electronic health record; EL: Expression Language.

The process of representing screening conditions is shown in Figure 1.

#### Screening Concept Definition

The screening conditions are composed of medical concepts and related constraints. Aiming at resolving the mismatch between screening concepts and data items in EHRs, a method was designed to define screening concepts from openEHR archetypes and templates, which are used to build data structures in EHRs. This way, screening conditions can be represented in a consistent way with EHRs to promote screening performance. For the management of screening concepts and the representation of complex constraints, relevant attributes need to be defined, as shown in Table 1.

**Table 1:** Definition of attributes of concepts.

Attribute	Description
Name	The name of a concept
Parent	Parent concept of the current concept, used to represent the hierarchical relationship among concepts
Path	The path of concepts as an identifier
Type	The data type of concepts, which decides allowed constraints
Unit	The unit of concepts, especially for laboratory test concepts
StartTime	The start time when an event happens, used to represent temporal constraints
EndTime	The end time when an event ends, used to represent temporal constraints
Value	The value of quantifiable concepts, such as the WBC^a^ count, or used to represent some constants such as the lower limit of blood pressure

^a^WBC: white blood cell.

OpenEHR archetypes define complete domain contents for clinical concepts, which are loosely bound to attributes and constraints for the consideration of generality and reusability in design. The template is a reasonable composition of one or several archetypes, which can be further constrained. Therefore, openEHR templates are used to directly define screening concepts. The rules for the definition of concepts are as follows:

1. A template corresponds to an archetype with detailed constraints, such as local optionality, default values. So, it is considered as a concept set. Each attribute node in the template is mapped to the subconcepts under the concept set.
2. When the attribute node in the template is mapped to a concept, the ontology name of the node is treated as the name of the concept. The data type of the node is used as the type of the concept, and the path of the node in the openEHR template is mapped to the path of the concept. For hierarchical relationships among different concepts, parent attributes can be identified by the preceding part of the children’s paths because these paths can be treated as identifiers of these attributes.
3. For DV_QUANTITY attributes, the attribute “units” can be mapped to the unit attribute of the concept.
4. For DV_CODED_TEXT attributes, the attribute “defining_code” can be mapped to subconcepts under this concept. This attribute definition can be from different terminology services. Therefore, for the same subconcept, there may exist several data items under the DV_CODED_TEXT attribute’s concept.
5. For other data types, relevant node items are just mapped to screening concepts according to step 2.

For the StartTime and EndTime of screening concepts, it is meaningless to extract from templates. Because these time attributes are related to specific clinical events, they can be specified to clinical concepts when screening patients. For example, the laboratory test may contain several concepts about time, such as result time, test time, and specimen receipt date and time. According to different application scenarios or the understanding of different researchers, the concept of a white blood cell (WBC) count can be bound to a different StartTime and EndTime.

#### Screening Concept Generation

Although some concepts can be defined according to rules directly from openEHR templates, there exist complex concepts and constraints that cannot be derived from these templates in practical cases, such as “white blood cell count continues to decrease in a specific duration after chemotherapy and radiation”. For the constraint “decrease,” no ready-made expression nor operators can represent it. In addition, chemotherapy is indicated by relevant drugs in general, such as altretamine, bendamustine, and azacytidine, instead of kept in EHRs directly. These expressions, such as chemotherapy, need to be specified by combining existing concepts and constraints.

OpenEHR EL is used to express complex constraints based on templates. Applying openEHR EL to generate customized concepts requires declaring variables, binding variables and data items in EHRs, and defining the logical expressions of simple concepts.

**Figure 2:**
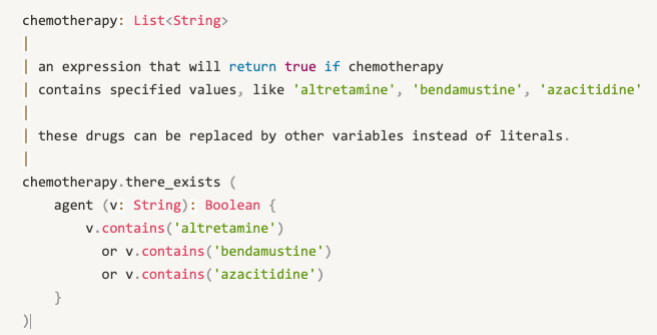
The representation of “derived concepts” chemotherapy.

To declare variables, openEHR EL supports variable declarations, assignments, and expressions. So, customized concepts can be generated in the form of variables. For example, chemotherapy can be generated, as shown in Figure 2.

The mapping between openEHR templates and concepts has been realized in the process of screening concept definition and the structure of an EHR comes from archetypes and templates. As a result, the derived concepts are customized and can be directly used as the variable in EL expression to realize implicit binding.

**Figure 3:**
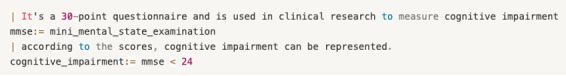
The representation of derived concepts, which is based on relational operators.

**Figure 4:**
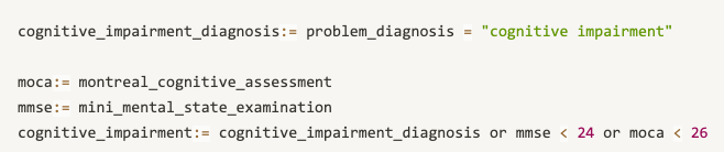
The representation of derived concepts by logical operators.

These customized concepts consist of logical expressions and defined concepts directly from templates. According to different types of constraints, the customized ways can be divided into three types:

1. The first way is to constrain defined concepts by arithmetic operators. For example, the body mass index (BMI) does not occur in EHRs directly. To screen patients by this condition, the BMI is defined as “BMI := weight/height^2”.
2. Another way is to generate customized concepts with relational operators. For example, cognitive impairment can be generated, as shown in Figure 3. The Mini-Mental State Exam (MMSE) is a widely used test of cognitive function. Any score of 24 or less (out of 30) indicates an abnormal cognition. This kind of knowledge can be an intuition for clinical researchers on stroke but is not identified by screening tools.
3. Complex customized concepts can be generated by combining arithmetic operators or condition chains by logical operators. Figure 4 gives an example of this. The cognitive impairment diagnosis concept can be defined directly from templates about problem/diagnosis. Two test scales are used to measure the different levels of cognitive impairment. By combining these three expressions, the new customized concept can help screen more eligible patients with high accuracy.

**Figure 5:**
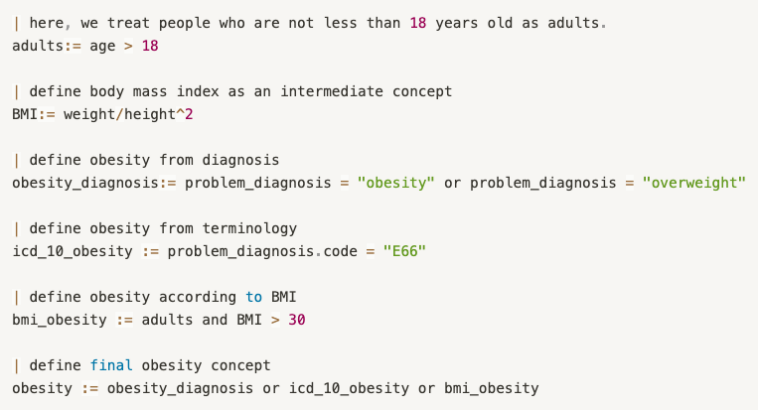
The representation of intermediate concepts for derived concepts.

There are other issues to consider in the generation of customized concepts. One of the issues is the generation of nested concepts. Because some concepts are complex, only simple concepts defined from templates cannot meet the requirements of clinical scenarios. In these cases, intermediate concepts need to be first generated, and then customized concepts are expressed based on these intermediate concepts. For example, although obesity/overweight can be defined in openEHR templates with a specific terminology from the International Classification of Diseases, Tenth Revision (ICD-10), numbered E66, some expressions can be treated as the same criteria semantically, as follows in Figure 5.

#### Constraints About Screening Concepts

Operators provided by openEHR EL meet the maximum requirements of representation to express existential, arithmetic, logical, and relational constraints. In some cases, it is a challenge to represent constraints about collections and time.

The screening conditions about collections are highly flexible. In general, knowledge engineers are required to define these constraints for specific clinical requirements. For different medical institutions, even different clinicians, the understanding of these constraints can be different. Therefore, according to collected screening conditions, some constraints are predefined for convenience, as shown in Table 2.

**Table 2:** Predefined constraints for collections.

Constraint name	EL^a^ expression	Parameter
First time	[concept].count()=1	Concept name
Stable	[concept].max()-[concept].min()<[value]	Concept name; self-defined threshold value for the comparison
Increase	[concept].last()>[concept].first()	Concept name
Decrease	[concept].last()<[concept].first()	Concept name

^a^EL: Expression Language.

To further constrain some concept set, the screening tool supports clinical research to self-define new constraints and edit existing constraints, in addition to using predefined constraints.

Allen et al [32] summarized 13 temporal representation patterns for comparing two events. They are represented by expressions in our tool, as shown in Table 3.

These temporal constraints cannot meet the requirements in some cases, for example, “The patient was treated with heparin within 48 hours.” According to the analysis about collected criteria, an “interval” attribute was introduced into our tool based on Allen et al's [32] patterns to represent the interval between different clinical events, for example, “diff([concept1].StartTime, [concept2].EndTime) [><=] Interval”.

**Figure 6:**
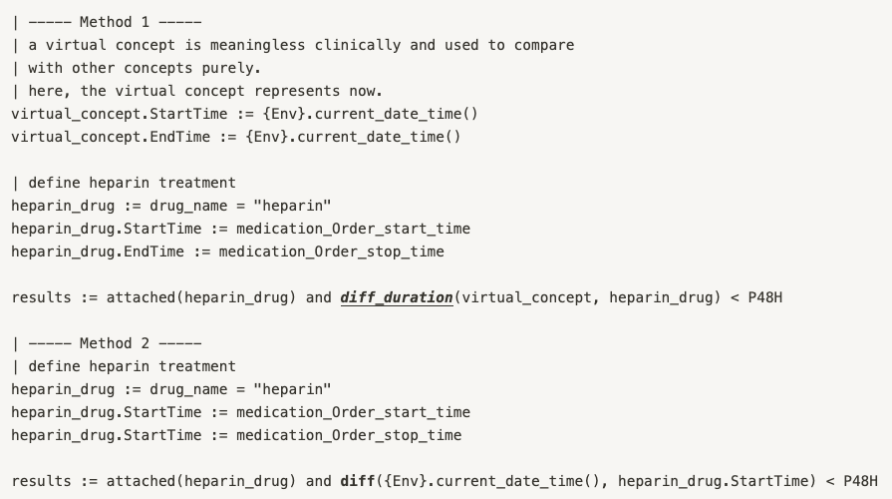
An example of temporal constraints.

By combining these extended constraints and the 13 patterns proposed by Allen et al [32], most screening conditions can be represented about temporal constraints. For example, “The patient was treated with heparin within 48 hours” can be represented in two ways, as shown in Figure 6.

**Table 3:** Representation of Allen temporal patterns.

Name	Expression	Description
Before	[concept1].StartTime<[concept1].EndTime<[concept2].StartTime<[concept2].EndTime	Concept1 occurs before Concept2.
Meets	[concept1].StartTime<[concept1].EndTime=[concept2].StartTime<[concept2].EndTime	Concept2 occurs at the end of Concept1.
Overlaps	[concept1].StartTime<[concept2].StartTime<[concept1].EndTime<[concept2].EndTime	Concept1 occurs before Concept2 and ends before Concept2.
Begins	[concept1].StartTime=[concept2].StartTime<[concept1].EndTime<[concept2].EndTime	Concept1 and Concept2 occur at the same time, and Concept1 ends first.
BegunBy	[concept1].StartTime=[concept2].StartTime<[concept2].EndTime<[concept1].EndTime	Concept1 and Concept2 occur at the same time, and Concept2 ends first.
During	[concept2].StartTime<[concept1].StartTime<[concept1].EndTime<[concept2].EndTime	Concept1 occurs after Concept2, and Concept1 ends before Concept2.
Contains	[concept1].StartTime<[concept2].StartTime<[concept2].EndTime<[concept1].EndTime	Concept1 occurs before Concept2, and Concept1 ends after Concept2.
Equals	[concept1].StartTime=[concept2].StartTime<[concept2].EndTime=[concept1].EndTime	Concept1 and Concept2 occur and end at the same time.
OverlappedBy	[concept2].StartTime<[concept1].StartTime<[concept2].EndTime<[concept1].EndTime	Concept1 occurs after Concept2, and Concept1 ends after Concept2.
Ends	[concept2].StartTime<[concept1].StartTime<[concept1].EndTime=[concept2].EndTime	Concept1 occurs after Concept2, and both end at the same time.
EndedBy	[concept1].StartTime<[concept2].StartTime<[concept2].EndTime=[concept1].EndTime	Concept1 occurs before Concept2, and both end at the same time.
MetBy	[concept2].StartTime<[concept2].EndTime=[concept1].StartTime<[concept1].EndTime	Concept1 occurs at the end of Concept2.
After	[concept2].StartTime<[concept2].EndTime<[concept1].StartTime<[concept1].EndTime	Concept2 occurs before Concept1.

### Execution of Screening Conditions

Different from the method where the screening expression and constraints are directly hard-coded, such as i2b2, ANother Tool for Language Recognition (ANTLR) [33] is used to parse openEHR EL expressions. In addition, a translator is implemented to transform the screening conditions into the underlying query language. The decoupling design uses the mechanism of openEHR two-level modeling and makes it easy to keep maintainability.

**Figure 7:**
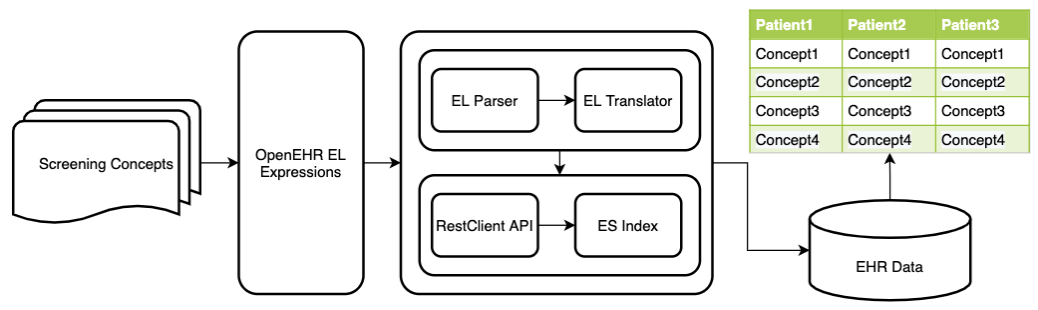
The architecture of query execution for screening conditions. API: Application Programming Interface; EHR: electronic health record; EL: Expression Language; ES: Elasticsearch.

Most openEHR-based CDRs are based on relational databases [19,20]. The data scattered in multiple tables make it unavoidable to bring multitable joins and subqueries. To improve query performance, this study decided to use a dedicated search engine to execute queries. ES can store, search, and analyze a large amount of data in a short time and can meet the performance requirements of patient screening as a distributed search engine. Similar to relational databases, each typed field can be indexed and queried. The architecture is designed for the execution of queries in Figure 7.

Data are stored as a document in ES, and the document's schema is defined by mapping. The index is similar to a database, and the type is similar to the table structure in a relational database. ES documents store data in the form of key–value pairs, and documents can also be used as class types of values to achieve hierarchical storage. To query EHR data in an index, this study proposed a method that maps openEHR templates to index structures.

**Figure 8:**
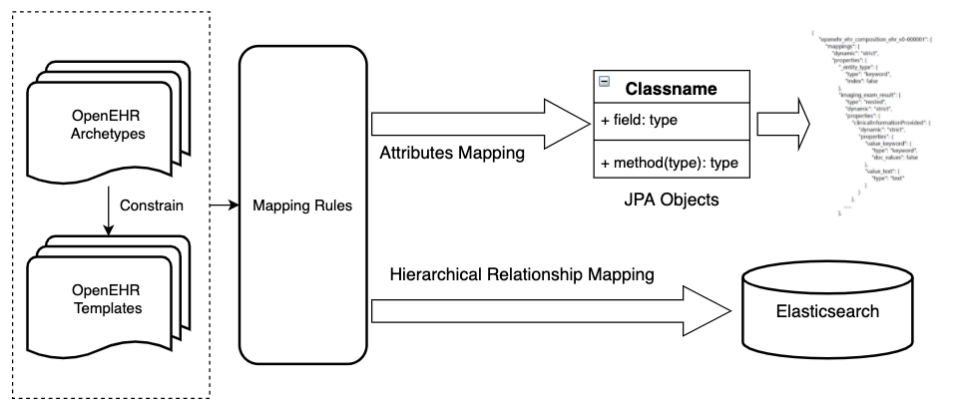
The flowchart of mapping between templates and ES. API: Application Programming Interface; EHR: electronic health record; ES: Elasticsearch; JPA: Java Persistence API.

Specifically, openEHR templates are used to generate corresponding entities conformed to the Java Persistence Application Programming Interface (API), or JPA [34], according to a series of rules. Then, mapping relationships between entities and indexes are described according to the ES-related annotations provided by a hibernate search [35]. Finally, a schema of the index structures is generated by using the existing hibernate search framework. The flowchart is shown in Figure 8.

#### Mapping Rule Definition

Mapping rules decide the detailed structure of the index, which plays a significant role in performance. Template index-mapping rules can be divided into several parts:

1. Data type mapping
2. Hierarchical relationship mapping
3. Naming strategies

A composition archetype and a corresponding template are required as a container to import other archetypes, including demographic, imaging examination, laboratory test, problem diagnosis, medication order, and procedure. The composition template is designed into a single index in ES. Corresponding to the definition of screening conditions, attribute nodes and the hierarchical relationship are the focus in the mapping process.

#### Data Type Mapping

The data types of openEHR template attributes are defined in the RM, and since each field of ES has a type, each RM needs to add corresponding field-type annotations to map to the corresponding entity object types. Table 4 shows the mapping relationships between commonly used data types and entities.

Field annotations decide not only the data types in ES but also the analyzers that carry out indexing and text processing used in these fields. All data types are mapped into three field types:

1. GenericField: Use a default field type and analyzer for the specific attribute type according to provided strategies by the hibernate search.
2. KeywordField: It only works for string fields whose value is treated as a single keyword. So, it is appropriate for terminology-constrained diagnosis, laboratory tests, imaging examination, etc.
3. FullTextField: Compared with KeywordField, data are treated as free text, and so this only works for string fields. The text is split into several tokens as an index. Here, for string fields in DV_CODED_TEXT and DV_TEXT, two kinds of field annotations are added so that they can be queried in different ways.

**Table 4:** Data type mapping rules for openEHR^a^.

Data type	Attribute	Field type	Field annotation
DV_BOOLEAN	value	Boolean	GenericField
DV_CODED_TEXT	value	String	KeywordField; FullTextField
	code	String	KeywordField
DV_COUNT	magnitude	Integer	GenericField
DV_DATE	dateTime	LocalDate	GenericField
DV_DATE_TIME	dateTime	LocalDate	GenericField
DV_DURATION	duration	Duration	GenericField
DV_IDENTIFIER	id	String	KeywordField
DV_QUANTITY	magnitude	Double	GenericField
	units	String	KeywordField
DV_TEXT	value	String	KeywordField; FullTextField
DV_URI	uri	URI	GenericField

^a^EHR: electronic health record.

#### Hierarchical Relationship Mapping

The composition template corresponds to only one index structure. The imported archetypes generate hierarchical relationships in this index. Attributes with basic data types, such as string, int, and date, can be indexed as value fields. These imported archetypes and underlying slotted archetypes and attributes of collection types are indexed as object fields. Several Java Persistence entities are built according to these archetypes. The detailed rules are as follows:

1. Each archetype corresponds to a master entity object that contains the indexed annotation.
2. Slotted archetype and collection-type attributes are mapped into a new embedded entity in the master entity. However, there exists a difference between these two types. A slotted archetype can be treated as an independent medical concept and is considered as a normal object field, which means all of its attributes are flatted during indexing. For an attribute of collection type, it is mostly attached to a major medical concept and cannot be used on its own. Therefore, it is mapped into a nested field type, which can keep the original structure in the archetypes.
3. Every generated entity is labeled with an ID field that uniquely identifies a document.
4. Every generated entity is also labeled with a timestamp field, which is treated as a major time marker for comparison, which can be specified during data integration.
5. Every field type defined in the openEHR RM is considered as an embedded entity.

#### Naming Strategies

1. The name of attributes in archetype data types is predefined according to Java field names. Particularly, the Java string field, which is annotated with two annotations, KeywordField and FullTextField, is mapped to two fields named “{java field name}_keyword” and “{java field name}_text”.
2. For attributes within entry archetypes directly, the names are defined with their textual name mentioned in the ontology section of archetypes. This text is joined with “_”. For unique identification in the index, every attribute's name is prefixed with the archetype concept name; for example, “Body site” in openEHR-EHR-EVALUATION.problem_diagnosis.v1 will be named with “problem_diagnosis_Body_site”.
3. For attributes within slotted and collection-type archetypes, the name is decided by two parts: one is these direct parent archetypes; the other is entry archetypes imported in the composition template. For multiple levels of archetypes, the name is provided recursively.

### Development of a Screening Tool

**Figure 9:**
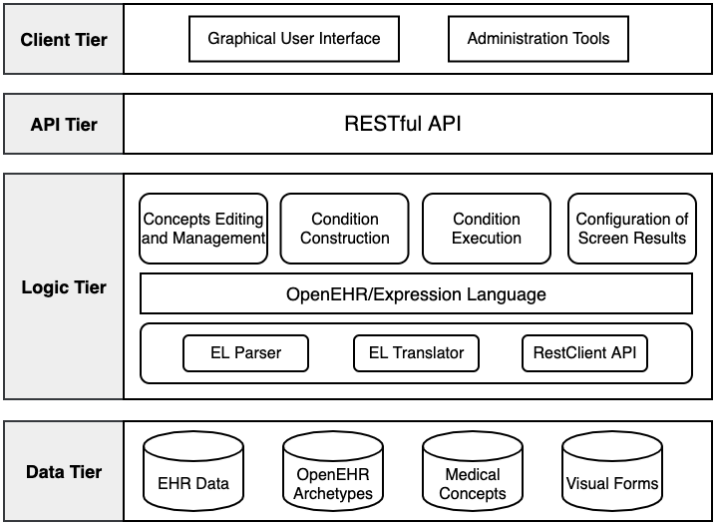
System architecture for the patient-screening tool. API: Application Programming Interface; EHR: electronic health record; EL: Expression Language.

Based on the proposed method above and requirements analysis, we developed a patient-screening tool on EHRs using openEHR. The system architecture is shown in Figure 9. Our tool uses a loosely coupled architecture where all modules are connected loosely so that they can be maintained and replaced more easily. To be specific, the system is mainly divided into three parts:

1. Concept editing and management: This is to realize the maintenance and management of screening concepts by definition and generation. Clinical researchers can edit and revise these concepts according to specific requirements.
2. Screening conditions’ construction/execution: An easy-to-operate visual interface is provided for users to edit screening conditions, and then restful APIs are used to execute queries in ES.
3. Results of screening configuration: Aimed at different data requirements, researchers can predefine specific data views in forms. This module makes it more convenient to get access to screening results by customized views.

#### Concept Editing and Management

The main function of this module is to manage screening concepts and to provide a concept generation function based on openEHR templates and openEHR EL so that users can quickly realize the mapping between screening concepts and EHR data.

**Figure 10:**
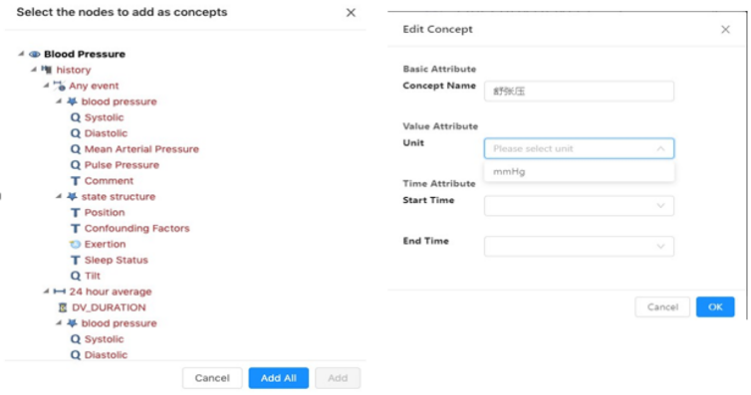
Editing concepts after selection in template.

OpenEHR templates used for EHRs are obtained from the template repository and parsed by tools provided by the openEHR community [36]. The screening concepts are generated based on the obtained templates. Since templates used by the electronic medical record system are for routine delivery of health care, part of them is not required for clinical research. So, these templates and related nodes can be selected according to real situations. Basic concepts can be defined from these templates, complex or derived concepts should be generated by the basic concepts, and constraints should be provided by openEHR EL, as shown in Figure 10.

#### Screening Conditions’ Construction/Execution

Screening conditions’ construction/execution is the core component of this screening tool, with which researchers formally construct screening conditions. Based on the screening conditions, a query is executed by calling the restful API provided by ES.

**Figure 11:**
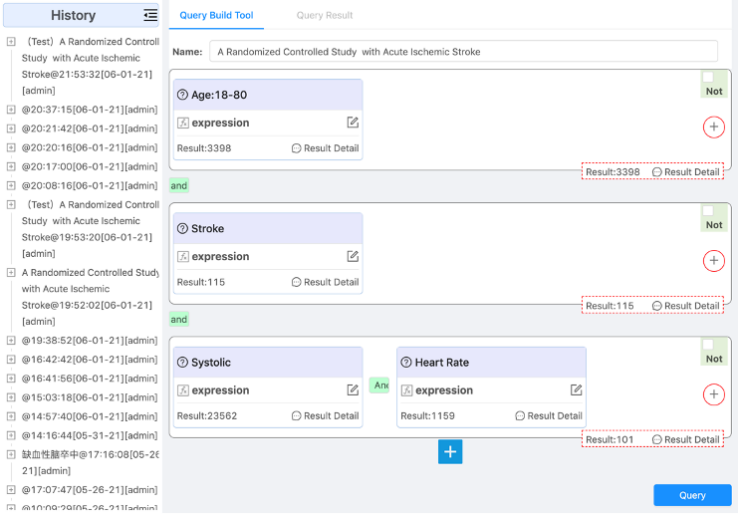
The user interface for construction of screening conditions.

A graphical editing interface is provided to support the construction of screening conditions. It is designed to organize components in hierarchical form to accomplish construction. Screening conditions can be divided into different groups corresponding to different visual components. Every screening concept is filled into a single group. Meaningful feedback is necessary because researchers do not know the exact data in EHRs. When constructing screening conditions, the number of results for the screening conditions in every group is immediately queried to support the revision of conditions. By default, groups are connected by logic conjunctions. Logical disjunction can be used within groups. For a single group for a screening concept, multiple constraints can be added by just dragging visual components filled with necessary information. Temporal constraint controls can be added between different groups. The user interface is shown in Figure 11.

**Figure 12:**
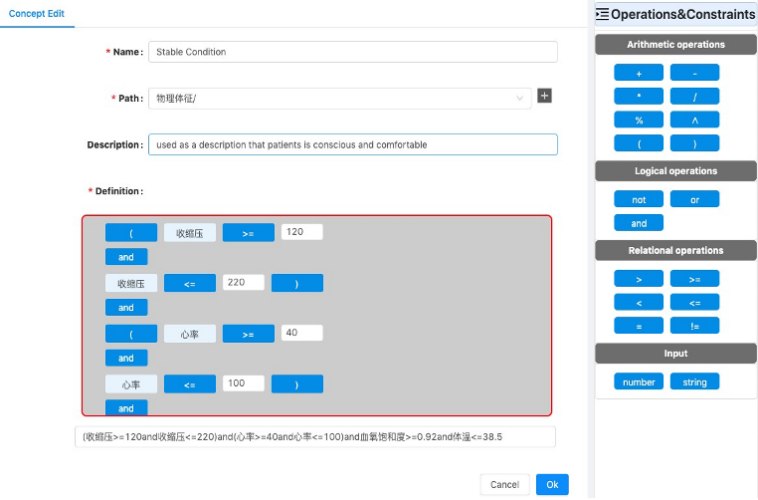
Derived concepts' generation.

Screening conditions contain complex collection and temporal constraints. Users can define customized visual components of constraints according to their own needs. Through the defined visual components, the corresponding openEHR EL expressions are generated to express complex conditions, as shown in Figure 12.

Screening conditions are constructed each time from scratch. It is unnecessary and time consuming because some conditions seem to be similar to a certain extent, such as conditions including the same concepts. So, our tool provides a function for saving constructed screening conditions for reuse after execution. Later, users can reuse the screening conditions without construction again. These screening conditions are saved in hierarchical form, and the required screening conditions are dragged and dropped at the corresponding level of the current conditions.

#### Results of Screening Configuration

The screening results’ viewing assists researchers to view the details of selected patients to further determine whether the patients meet the requirements. Some research platforms usually use the case report form (CRF) to record patient data. Our tool relies on a developed CRF generation service to help researchers customize patient result forms so that they can view information according to specific needs.

## Results

### Experiments for Representation

An experiment was carried out in an openEHR CDR based on a grade A tertiary hospital in China. In total, 500 sentences in the collected 389 clinical trials were randomly selected to analyze and evaluate the effectiveness of the proposed tool. A clinician and two information technology personnel joined the experiment. The clinician was responsible for providing medical domain knowledge and giving reliable proof when faced with different opinions. One of the information technology personnel took charge of the issues about openEHR, and the other was the core developer of the tool.

In this CDR, more than 30,000 concepts were directly defined from 34 openEHR templates. These concepts were used to represent 500 eligible criteria mentioned above. In these conditions, 589 concepts were found. Among the concepts found, 513 (87.1%) concepts could be represented and 471 concepts could be directly defined from templates. In addition, 42 concepts needed to be generated by the configurable operation, and there still existed a challenge to represent 76 concepts. Table 5 shows the part of configurated concepts.

At the same time, our experiment was also carried out in an i2b2 web client to figure out the differences between our tool and the client. By comparing the provided query functions, the differences are given in Table 6 (Y means yes, and N means no).

**Table 5:** Part of configurated concepts.

Name	Descriptive expression	Mentioned times (n)
Course of a disease	current_date_time() - encounter.StartTime	78
Stable condition	(Systolic blood pressure≥120 and systolic blood pressure≤220) and (40≤heart rate≤100) and blood oxygen saturation≥92% and body temperature≤38.5	10
In good spirits	27<mmse^a^<30	2
Dual-antiplatelet therapy	attached(aspirin) or attached(clopidogrel)	1
Cognition impairment	cognitive_impairment_diagnosis or mmse<24 or moca^b^<26	11
Obesity/overweight	obesity_diagnosis or icd_10_obesity or bmi_obesity	2
Psychotropic drugs	attached(sulpiride) or attached(risperidone)	2

^a^mmse: Mini-Mental State Exam.

^b^moca: Montreal Cognitive Assessment.

**Table 6:** Comparison with the i2b2^a^ web client.

Constraint support	Details	Our tool	i2b2	Example
Exist	—	Y	Y	Patients with stroke
Relational	—	Y	Y	Patients aged 30-80 years
Logical	—	Y	Y	Cerebral infarction or cerebral hemorrhage and subarachnoid hemorrhage
Temporal	Duration	Y	N	Operation time <1 h or >3 h
	Interval among different clinical events	Y	Y	Antiplatelet drugs within 2 weeks before surgery
	Interval related to a single clinical event	Y	N	Treated with botulinum toxin injection within 6 months
Collection	Count	Y	Y	First onset
	Complex computation on collections	N	N	Average treatment time less than 1 h per week
Self-defined constraint	—	Y	N	WBC^b^ count decreasing
Self-defined concept	—	Y	N	Stable condition

^a^i2b2: Informatics for Integrating Biology & the Bedside.

^b^WBC: white blood cell.

### Evaluation for Performance

**Figure 13:**
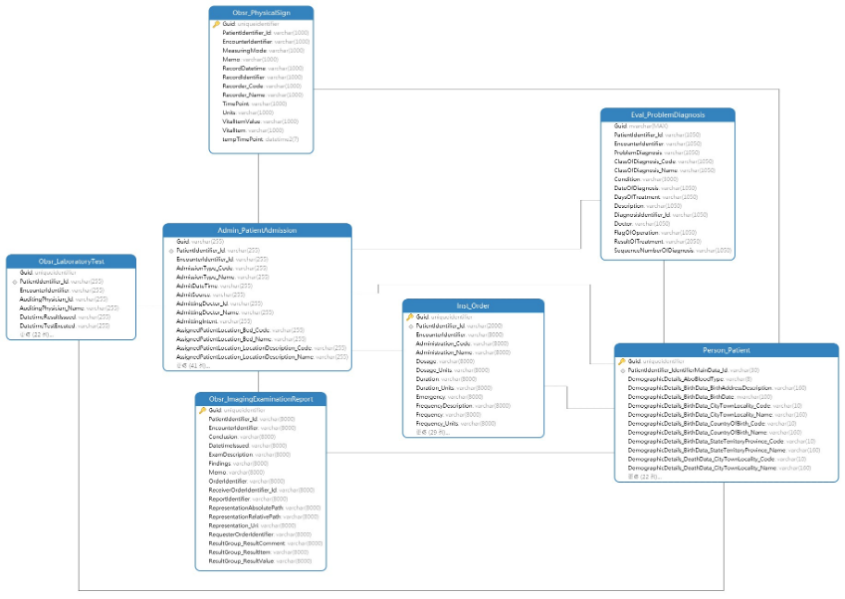
Database schemas by archetype relational mapping.

**Figure 14:**
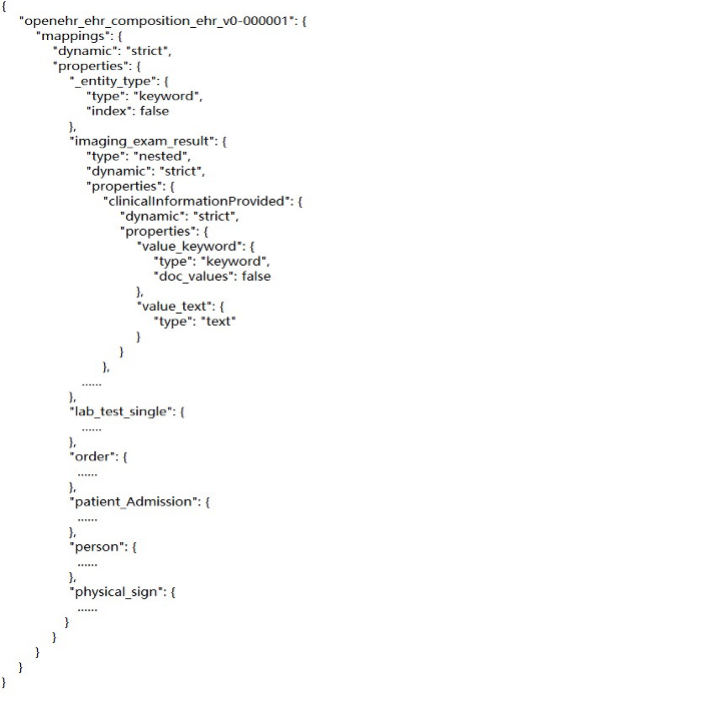
Index structure for the openEHR template. EHR: electronic health record.

An evaluation was performed to validate the query performance of our method. The used data are from the hospital mentioned before. EHRs store all the information generated during the routine delivery of health care, and part of them is about management and charge information, which are not required by clinical research. So, a total of seven archetypes and related templates were selected, including demographics, examinations, and laboratory tests. The selected templates are shown in Table 7. Considering the sensitivity of data and the complexity of data integration, only data related to cerebrovascular diseases were extracted, including 95,226 records of demographics, 449,880 records of admission, 4,239,454 records of physical sign information, 5,832,990 records of laboratory tests, 12,966,659 records of order information, 158,240 records of diagnostic information, and 176,798 records of imaging examination. The data storage structure was generated based on archetype relational mapping [19] and our template index-mapping method and is shown in Figure 13 and Figure 14, respectively.

**Table 7:** OpenEHR^a^ archetypes for EHR structures.

Name	Archetype
Person	openEHR-EHR-ADMIN_ENTRY.person.v1
Patient admission	openEHR-EHR-ADMIN_ENTRY.Patient_Admission.v2
Laboratory test	openEHR-EHR-OBSERVATION.lab_test_single.v1
Order	openEHR-EHR-INSTRUCTION.order.v1
Imaging examination	openEHR-EHR-OBSERVATION.Imaging_examination_report.v2
Physical sign	openEHR-EHR-OBSERVATION.physical_sign.v1
Problem diagnosis	openEHR-EHR-EVALUATION.problem_diagnosis.v1

^a^EHR: electronic health record.

The test cases were executed in Windows 10 with 16GB RAM and an Inter(R) Core (TM) i5-4590 CPU including Microsoft SQL Server 2014-12.0.2269.0 and Elasticsearch-7.11.1.

Our study selected six screening conditions as test cases, and each test case was tested five times in two test environments to eliminate accidental errors. The execution time of the screening conditions was separated into two parts: translation time of expressions and query time in underlying persistence layers. The selected six test cases are shown in Table 8.

**Figure 15:**
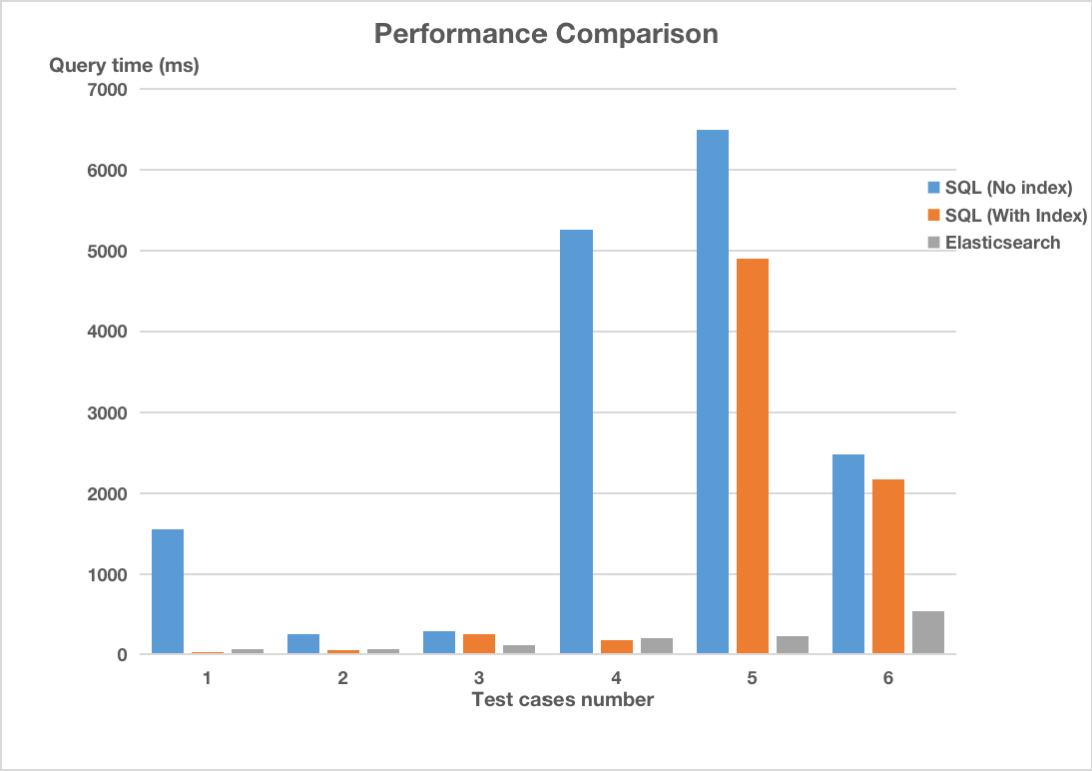
The performance comparison results between two methods. SQL: Structured Query Language.

The test results of these test cases are shown in Table 9 (for archetype relational mapping) and Table 10 (for template index mapping). Their comparison result is shown in Figure 15.

**Table 8:** Test cases for performance evaluation.

Test case	Condition	Description
Query1	Patients with evacuation of intracerebral hematoma	Occur in a single table, no join operation required
Query2	Female patients between 20 and 60 years	Occur in a single table, no join operation required
Query3	60-70-year-old female patients diagnosed with cerebral hemorrhage or cerebral infarction	Occur in two table, join operation between two tables required
Query4	Women between 60 and 70 years diagnosed with cerebral hemorrhage or cerebral infarction taking aspirin	Occur in three tables, join among three tables required
Query5	Female patients between 60 and 70 years diagnosed with cerebral hemorrhage or cerebral infarction undergoing a WBC^a^ laboratory test and taking aspirin	Occur in four table, join operation among four tables required
Query6	The last WBC count more than 10×10^9^/L	Sort and aggregation operation probably required

^a^WBC: white blood cell.

**Table 9:** Test results for archetype relational mapping.

ID	SQL^a^ execution time without index (ms)	SQL execution time with index (ms)	Number of results (n patients)
Query1	1547	30	40
Query2	256	59	8570
Query3	284	257	1536
Query4	5253	183	154
Query5	6497	4893	106
Query6	2484	2193	14,583

^a^SQL: Structured Query Language.

**Table 10:** Test results for template index mapping.

ID	Translation time of EL^a^ (ms)	Query time (ms)	Total time (ms)	Number of results (n patients)
Query1	46	22	68	40
Query2	62	10	72	8570
Query3	62	49	111	1536
Query4	136	64	200	154
Query5	153	75	228	106
Query6	169	364	533	14,583

^a^EL: Expression Language.

The test results show that the number of screening results obtained by the two methods is the same, and it can be considered that the screening tool proposed can correctly obtain screening results.

Considering query performance, in general, SQL queries with an index outperform SQL queries without an index. For queries using SQL (Query1 and Query2) without joins, the more data in the table, the longer the execution time. In addition, the query time increases rapidly (Query3, Query4, and Query5) during joins among three tables or even four tables.

With regard to execution using ES, it does not show obvious advantages among queries without joins, such as Query1 and Query2. However, for queries that are related to joins, ES outperforms SQL queries because the patient data are indexed with a single document, there is no need for joins among different documents, and the performance is more stable. At the same time, there exists only a small gap between the execution time of the search engine and the translation time of screening conditions. Considering the volume of EHR data on disks and the translation running in memory, the translation time has a serious influence on query performance. Therefore, there is still significant room for further improvement.

In comparison between the two methods, the screening execution method in our study showed better performance than the SQL-based method, especially in the screening context of multitable joins (Query4 and Query5).

## Discussion

### Principal Results

We proposed a patient-screening tool using openEHR to transform screening conditions into expressions for queries on EHRs. The tool is designed to support queries on EHRs directly within a local context. To sum up, our tool has the following features:

- First, the tool supports definition and generation from openEHR archetypes and templates. These concepts can be simple concepts and derived concepts. In previous studies, many tools have just provided a fixed-concept set based on some terminologies. Although a related study is proposed to extend concepts, these extended concepts can be only added by SQL expressions, which is a big challenge for clinical researchers.
- Second, the tool improves the performance of screening compared with SQL-based methods. With the continuously increasing data in EHRs, there is a serious bottleneck for queries in these situations. Our method proposes an implementation of openEHR AMs to promote query enhancement based on ES. It is worth taking as a reference to design other query tools not limited to clinical research.
- Third, the tool provides a promising solution for secondary use of EHRs in clinical research for the openEHR community. To the best of our knowledge, there is no such tool based on openEHR. Our study shows that although openEHR specifications are mostly designed for the EHR environment, they can be used for clinical research in a way proposed in this study. Although our method is proposed within the context of openEHR, other information models can be translated into openEHR information models, which is proved by previous research [37]. In this way, our method can be used in these information models.

### Ability of Representing Complex Concepts

According to the results of experiments for representation, 87.1% of concepts can be represented by our method. In addition, 76 concepts (12.9%) were not expressed successfully because of the complexity of screening conditions. Some reasons are as follows:

- First, no appropriate archetypes or templates can be used to generate these concepts. In other words, the necessary basic concepts are not covered by the EHR information models. In addition, the concepts that occurred in conditions are not recognizable by our templates due to differences. With this kind of issue, more archetypes and templates need to be encouraged to be developed for specific requirements. In addition, more local knowledge should be introduced into templates.
- Second, some concepts do not occur in a structured way. For example, “severe coronary stenosis” is mostly recorded in the description of imaging examination. In these situations, concepts cannot be defined from openEHR templates directly for screening. This limitation can be solved in two ways. One is to do what i2b2 does. New concepts can be extracted from medical texts with natural language processing (NLP), and a mapping relationship can be built between these new concepts and original text for backtrack during querying. Another way is to process these medical texts independently, and new strategies can be proposed to query texts together with structured data.
- Third, some concepts mentioned in these conditions are coarse grained and fuzzy. It is difficult to define a comprehensive expression to meet all requirements for all queries because of different knowledge backgrounds and considerations. For example, in the condition “patients diagnosed with the diseases that may lead to dysphagia,” it is difficult to represent ”the diseases.“ The definition of ”the diseases“ is general involving many concepts. Diseases leading to dysphagia can be of different types, including brain/nervous system diseases, muscle diseases, and esophageal diseases. The results of the definition can be a big list. In addition, different departments focus on different types of diseases. For example, stroke, Parkinson's disease, and other brain/nervous system diseases are considered in psychiatry departments. Oncology departments tend to consider esophageal carcinoma when finding dysphagia. The general description of concepts in screening conditions is common, and they play a significant role in hindering accurate querying.

### Limitations and Future Directions

Visual editing tools can reduce the burden of researchers, but they still require a certain amount of manual work. For example, such tools provide some modules such as reusing existing conditions for convenience. Developing new screening conditions from scratch is still inevitable, especially for complex conditions and conditions that have not been used before. With the continuous development of NLP technology, screening conditions in free text can be automatically converted into executable queries, such as SQL or openEHR EL expressions in this paper.

Stubbs et al [38] proposed the task of patient screening using EHRs. Some contestants [39,40] used text data to determine which patients meet the criteria by using rule-based methods, neural networks, etc. Some studies [41-44] have transformed conditions into a computer-interpretable format with information extraction. Criteria2Query [45] provides a natural language interface to help find eligible patients. In addition, some text-to-SQL methods are proposed to execute queries on EHRs [46]. This reduces the workload by predicting the SQL query for a given condition about a database.

To some extent, these studies greatly relieve the burden of researchers by allowing familiar way of humans to construct queries. Their work reduces the extensive interaction issues with systems/databases or administrators. However, the end-to-end process hinders manual participation, considering extremely complicated conditions. Meanwhile, for concept mismatch between conditions and EHR data, they do not provide an available solution.

Considering the advantages of NLP technology and the method proposed in this paper, the future direction for us is to combine machine learning methods, rule-based methods, and engineering to improve queries on EHR. Their combination will outperform any single method.

### Conclusions

In this paper, we developed a patient-screening tool for clinical research using openEHR. The tool helps solve concept mismatch, especially for derived concepts. The use of ES improves query performance compared with SQL-based methods. The tool is applied to stroke-related clinical research and shows promise. Moreover, we demonstrated a promising solution for secondary use of EHR data using openEHR. In the future, we will enhance the tool by leveraging NLP techniques to enable automatic query formulation for simple and derived concepts to further reduce the burden of researchers.

## Abbreviations

AM: archetype model
ANTLR: ANother Tool for Language Recognition
API: Application Programming Interface
CDR: clinical data repository
ChiCTR: Chinese Clinical Trial Registry
CRF: case report form
EHR: electronic health record
EL: Expression Language
ES: Elasticsearch
i2b2: Informatics for Integrating Biology & the Bedside
ICD-10: International Classification of Diseases, Tenth Revision
JPA: Java Persistence API
MMSE: Mini-Mental State Exam
MoCA: Montreal Cognitive Assessment
NLP: natural language processing
RM: reference model
SQL: Structured Query Language
